# Local ecological factors, not interference competition, drive the foundress number of two species of fig wasp sharing *Ficus septica* figs

**DOI:** 10.1371/journal.pone.0290439

**Published:** 2024-01-02

**Authors:** Bruno Di Giusto, Anthony Bain

**Affiliations:** 1 Journalism and Mass Communication Program, International College, Ming Chuan University, Taipei, Taiwan; 2 Department of Biological Sciences, National Sun Yat-Sen University, Kaohsiung, Taiwan; 3 International Ph.D. Program for Science, National Sun Yat-Sen University, Kaohsiung, Taiwan; National Institute of Agricultural Research - INRA, MOROCCO

## Abstract

Recent studies have challenged assumptions about the classic fig-fig wasp pollination mutualism model, suggesting that further investigation into the receptive phase of fig development is needed. This study assessed the pollination mechanisms of *Ficus septica* in southern Taiwan and identified two species of wasps as the primary pollinators. Machine learning was used to identify and rank the factors that explain the relative abundance of these wasps. The two wasp species showed the highest level of cohabitation ever reported in the literature, with three-quarters of the figs containing multiple foundresses. The study also reported re-emerged foundresses and a 10% ratio of pollinated figs without foundresses. Local factors, such as the sampling period and tree identity, were the best predictors of the presence and number of each foundress species, with fig size also affecting the number of foundresses. The study highlights the variability in pollinator abundance between figs, crops, and trees. It also shows that the local environment of the trees and the availability of figs are crucial factors in determining which figs the pollinator wasps choose. These findings challenge assumptions about the classic mutualism model and suggest that long-term surveys are needed to estimate the relative contributions of each partner and provide data for evolutionary and ecological models. This study also provides valuable insights into the factors that affect the abundance and interactions of pollinator wasps during the receptive phase of fig development, with implications for understanding the behaviour of pollinating wasps and advancing our knowledge of population dynamics in *Ficus* species.

## Introduction

The relationship between *Ficus* trees (Moraceae) and pollinating fig wasps (Hymenoptera: Agaonidae) is an obligate nursery pollination mutualism [1]. Fig trees provide oviposition and nursery sites for the wasps in exchange for pollination service. The flowers of the *Ficus* trees are enclosed in an involuted structure, the fig, and difficult to reach for most organisms but their specific pollinators, the fig wasps. When the female flowers inside the fig are ready to receive pollen, the fig emits a scent that attracts its specific pollinator [2]. As the fig wasp enters the fig through the narrow entrance called ostiole, it loses its wings and most of its antennae. Once in the fig, the fig wasp lays eggs in the ovule of the flowers, simultaneously smearing pollen from its natal fig on the style of other fig flowers [1]. A few weeks later, pollinated flowers produce seeds, while oviposited flowers produce wasp offspring ready to fly away and begin a new lifecycle. Only female wasps pollinate. Indeed, the wingless male wasps sole role is to fertilise the females and dig a way out of the fig for them; afterwards, they die. Estimated to be about 75 million years old, the relationship between *Ficus* trees and their pollinating wasps displays one of the most impressive and intricate levels of coevolution among living organisms [3].

It has long been assumed that this mutualism was working similarly among the 700 to 800 species within the *Ficus* genus: One species of fig wasp enters the figs of one species of *Ficus* (one-to-one rule), pollinates and lays eggs, then dies in the fig. The first observations breaking this rule came from Africa [4] and Central America [5] but were considered exceptions as they only concerned a few fig species. Nevertheless, since the beginning of the 21st century, cases of pollination by multispecific pollinators have shaken the homogeneity of the fig–fig wasp model. Observations of multiple pollinating wasp species sharing the same *Ficus* species have increased in numbers in Asia [6–8], Africa [9], and America [10, 11]. The current consensus is that the *Ficus* trees with the most extensive distribution (e.g., *F*. *racemosa*, distributed from India to Australia [12]) are often associated with more than one allopatric pollinator species.

Like the one-to-one rule, it has been traditionally assumed that foundresses die inside the first fig they enter as part of the *Ficus* pollination process. Once inside the fig, the female pollinating wasps are renamed foundresses as they found a new generation of insects after oviposition. The general belief was that foundresses, already maimed while crawling through the ostiole, perished in the fig after laying their eggs. However, re-emergence and secondary pollination in neighbouring figs are not uncommon, especially in Asian species (see review in [13]), despite being overlooked for a long time [1]. Secondary pollination affects many aspects of the fig–fig wasp mutualism as it may increase the genetic diversity of the wasp offspring [14], the number of pollinated figs [15, 16], and the pollination rate within figs [13]. If re-emergence is a more common trait than previously thought [13], it may offer new insights into our understanding of the fig wasp sex ratio [14] and its evolutionary significance on the mutualism stability [17, 18]. Two other common assumptions about *Ficus* pollination are that the number of foundresses within a single fig is low [19, 20] and that foundresses strongly bias their egg-laying towards more females [14].

Nevertheless, the documentation about the number of foundresses in figs comes from studies about fig wasp sex ratio. These studies have mainly focused on the number of produced fig wasp offspring during the wasp emergence phase. They have yet to give proper attention to the initial number of foundresses during the receptive (or pollination) stage. Recent findings showed that foundresses may sometimes gather in great numbers within a single fig [21, 22] and that the presence of one or several initial foundresses modifies the behaviour of the succeeding foundresses, which affects clutch size, oviposition sites, and sex ratio [22, 23 but see review in 14].

Interestingly, while the number of foundresses is such a critical feature of the fig-fig wasp mutualism, very few studies have focused on its variation within figs, across trees and time, and on the intrinsic and extrinsic factors responsible for it. Once again, an assumption is made: after mating in their natal figs, female pollinators disperse and randomly enter any receptive figs in their environment to start a new cycle. Is choosing to enter a receptive fig truly random if this fig has already been pollinated and oviposited, as pollination and oviposition will affect the reproductive success of any following foundress? Greeff & Newman [14] showed that the distributions of *Platyscapa awekei* in the crops of *F*. *salicifolia* were not different from a Poisson distribution, while others were clumped (negative binomial distribution). The authors suggested that pollinators released nearby the receptive figs and prevailing winds in the area could be responsible for the variation in lambda parameters across successive days and thus, clumped distributions. In contrast, Greeff et al. [22] showed that, among the three species sharing the figs of *F*. *sycomorus*, *Ceratosolen galili* and *C*. *arabicus* share figs less frequently than random. While this trait may result from active avoidance, the authors suggested that the fact that the two species fly at different times of the day may also explain this pattern.

These assumptions (one-to-one rule, no re-emergence, low foundress number, random settlement) mean we feed oversimplified data into our theoretical models. It also implies that the necessary data are overlooked, sparse, or incomplete in the literature. More observations in the natural environment are required to answer new questions, such as: What number and identity of foundresses result in the highest pollination rate? When two pollinating wasp species are observed on a single *Ficus* species, are they sharing figs? Does fig sharing involve direct, indirect, or no interaction between different fig wasp species? Does the presence of a foundress affect the behaviour of a fig wasp, whether conspecific or heterospecific, trying to enter the same fig?

As stated before, the fig phase of receptivity and fig visitation has been overlooked relative to the phase of wasp emergence. Figs may remain receptive over several weeks in the absence of wasp visitation (two to three weeks in *F*. *carica* and *aurea* [24] and two to four weeks in *F*. *hispida* and *F*. *exasperata* [25]). Further, receptivity stops when a “sufficient” number of pollinators enter the fig [24, 25]. When the receptivity ceases, the fig swells, the ostiole closes, and, at least in *F*. *hispida*, the composition of the odour bouquet that attracts the fig wasps drastically changes, signalling the end of receptivity [26]. To our knowledge, only Khadari et al. [24] have examined the impact of foundresses on the duration of fig receptivity. They found that figs stopped being receptive less than two days after being visited by three pollinators and up to four days after the visitation of a sole visitor. While these observations can provide insights into fig receptivity, how generalisable these results are remains an open question.

In terms of wasp oviposition, the above data suggest that the number of foundresses and, thus, the pollination ratio will restrict the windows of receptivity of the figs. Studies also demonstrated that wasp oviposition might be limited by (1) the number of fig ovules [27, 28], (2) the number of foundresses [23, 27–31], and (3) the clutch sizes [29, 32]. In response to such limitations, foundresses may exhibit behavioural adaptations allowing them to optimise their reproduction. For example, interference competition among *Platyscapa awekei* results from constraints in oviposition sites in monoecious *F*. *salicifolia* [27]. Larger foundresses aggressively hamper smaller ones from laying eggs while ovipositing themselves. In *F*. *salicifolia* figs, female wasps compete for both the number of eggs they can deposit and unused oviposition sites [27]. On *Ficus altissima* in southern China, conspecific females of the cheater *Eupristina* sp. and of the pollinator, *Eupristina altissima*, attack each other before entering figs and throw the loser of the fight off the fig [31]. However, they avoided fighting against other species entering the same fig. Dunn et al. [28] reported lethal competition in an undescribed *Pegoscapus* pollinating *F*. *citrifolia* in south-eastern Brazil. Aggression was only triggered once a fig wasp started to oviposit, with the first female to lay eggs decapitating other females in its vicinity. On the other hand, the foundresses of *Pegoscapus tonduzi* show little competition, maybe due to the low number of foundresses per flower in the figs of *F*. *citrifolia* in Panama [28]. The fact that *F*. *citrifolia* figs contain far more ovules than a female pollinator can fill and that some female wasps choose not to lay all the eggs they carry may explain this lack of interference.

Finally, *Ficus* species pollinated by several species of wasps offer various patterns of interaction. For example, in southern China, the cheater *Eupristina* sp. avoided confrontation with the pollinator, *Eupristina altissima*, and waited before following the pollinator inside the fig [31]. Other heterospecific pollinating wasps seem to avoid each other on *F*. *burkei* in South Africa [9] and *F*. *mexiae* in Brazil [11]. Furthermore, Cornille et al. [9] showed that in South African *F*. *natalensis*, competitive avoidance resulted in fewer figs (15%) sharing foundresses of different wasp species. Similarly, Greeff et al. [22] found that, among the three species sharing the figs of *F*. *sycomorus*, *Ceratosolen galili* and *C*. *arabicus* shared figs less frequently than expected by chance. While this trait may result from active avoidance, the authors suggested that temporal separation (the two species fly at different times of the day) may also explain this pattern.

Finally, Dunn et al. [33] proposed that fig wasps have no time for fig selection due to their short lifespan: they enter the first available fig they found. However, the cost associated with this absence of choice is a lower number of available ovules or a lesser quality oviposition site in an already pollinated fig compared to a receptive “virgin” fig (unvisited fig) [32]. In addition, re-emergence behaviour and secondary pollination could provide a significant evolutionary and ecological advantage to the individuals using it over others that do not, as male eggs laid in an already oviposited fig will increase the fitness of the re-emerging foundress. Thus, we expect female pollinators to be pickier than re-emerging foundresses over “virgin” figs. While observations of random settlement [29] support the assumption of no fig selection by the fig wasps, other observations of foundress either clumping together (if these observations are not extremely rare events) [21, 22, 29] or avoiding other foundresses [9, 22] do not. This assumption of no fig selection may then also require a re-evaluation.

**Fig 1:**
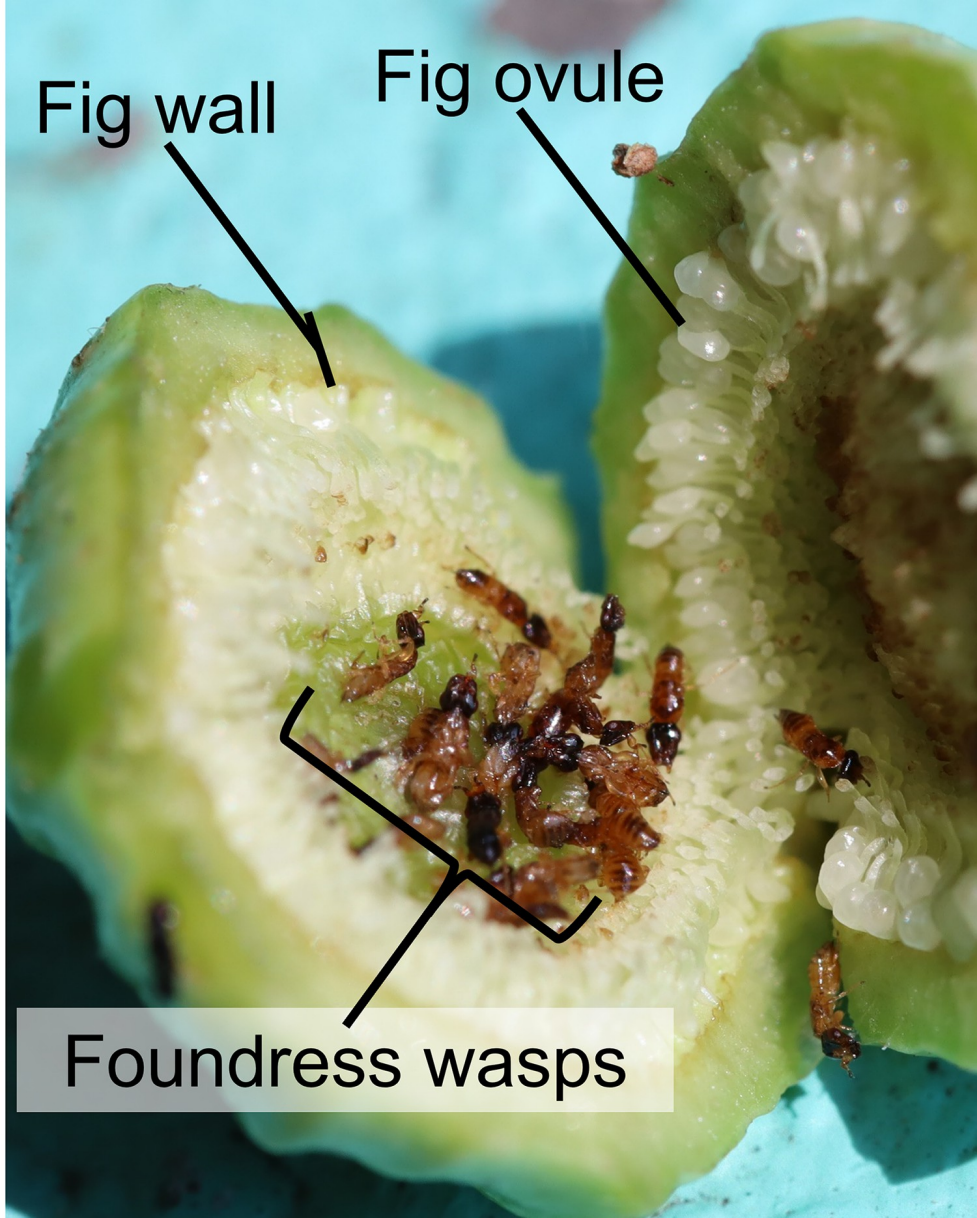
Foundresses pollinating wasps in an opened receptive fig of *F*. *septica*.

With all these unchecked aspects of the *Ficus* pollination and pollinator behaviour in mind, we assessed the pollination patterns of *Ficus septica*, a dioecious fig tree with an extensive distribution, in Southern Taiwan. In this area, two species of pollinators have been found in the figs of *F*. *septica* [6, 7]. Intriguingly, some crops of *F*. *septica* present an astonishing number of foundresses relative to other *Ficus* (Fig 1). The occurrence of such an event, too high to be a simple aberrance, prompted us to further explore the fig-fig wasp interaction in this fig tree. Then, focusing on the receptive phase of these figs and the number of foundresses, we used a longitudinal survey over two years to answer the following questions: (1) How many foundresses, on average, pollinate the figs of *F*. *septica*? (2) Does this average number vary across figs, trees, and time? (3) How do foundress numbers vary locally? Among trees? Or within a tree? (4) Do wasp species exclude each other or share the same fig? If yes, in which proportion? By gathering information about the pollination of *F*. *septica* over a long time, we hope to gain insights into how foundresses choose a fig to oviposit and the role of ecological factors on their local abundance.

## Materials and methods

### Study species

The fig tree *Ficus septica* is a gynodioecious species (figs of "male" trees generate pollen and wasps, whereas figs of "female" trees only generate seeds) distributed from the Ryukyu Islands to the North of Australia [34]. In contrast with most *Ficus* species, several species of Agaonid fig wasps, all belonging to the genus *Ceratosolen*, pollinate *F*. *septica* in Taiwan [6] and in the Philippines [7]. In Kaohsiung, two different wasps, a black one and an orange one, pollinate the figs of *F*. *septica*. Previous nuclear and mitochondrial analyses showed that the two wasps fall neatly into two different clades, indicating that they are two valid species [7] rather than colour variants of the same species. The orange species is most likely *Ceratosolen jucundus*, which was recently upgraded to species rank [7]. The other pollinating wasp species described as living on *F*. *septica* is *C*. *bisulcatus* from Java Island [35]. However, the colour description of *C*. *bisulcatus* does not match the fig wasps in this study. As no morphological or bar-coding identification was carried out during this study, we will refer to each wasp species as the orange and black morphospecies.

During the fig growing periods, branches continuously grow new figs, which means that figs are often at different stages of development (S1 Fig). However, despite the asynchronous nature of *F*. *septica* crops, female wasps emerging from their natal fig have a very low chance of finding a receptive fig on their natal tree. Only on a few male trees, both receptive and emerging figs have been observed on the same tree (Bain, pers. obs.).

### Survey

Between December 2018 and March 2021, 17 wild *F*. *septica* trees (ten males and seven females) growing in the National Sun Yat-Sen University (NSYSU) Campus, Kaohsiung, Taiwan, were monitored (no specific permit is necessary for NSYSU scholars to work on campus trees). Being wild, these trees exhibit a heterogenous range of shapes and likely ages, and irregular spacing with other trees and/or human structures. Approximately every two weeks and for 39 censuses, we collected, when possible, at least ten figs looking recently pollinated.

**Fig 2:**
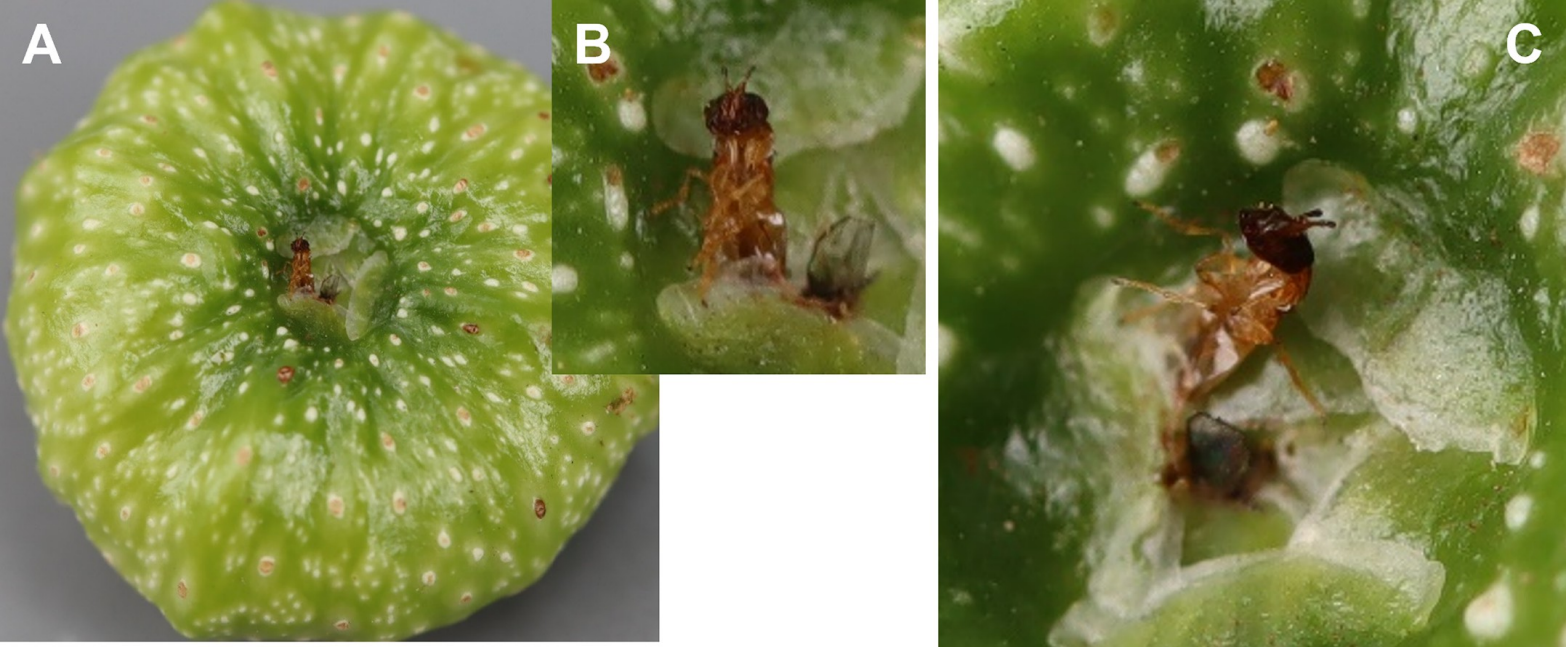
Foundress exiting a pollinated fig. The ostiole still exhibits the shiny integument of a receptive fig (A). The enlarged view shows an orange foundress exiting the ostiole and another pollinator entering the integument (B). An alternative view from another angle shows the detail of the integument (C). The appearance of the integument scales (smoothly stacked vs. crumpled) may indicate whether the figs have already been pollinated or not.

Recently pollinated figs can be recognised by their softness to the touch, shiny green integument (Fig 2A and S1 Fig), and unclosed ostiole (the opening through which flowers can be accessed). However, a few hours after pollination, the figs become turgescent and harden as the interfloral phase begins; this limits to one day, in the most appropriate conditions, the time when these figs can be collected.

Depending on availability, the ten figs were collected from a single tree. As fig size may be used as a proxy of flowers number (Wang et al., 2014), we took for each fig two measures of the diameter (the largest value (Diam1) and smallest value (Diam2)) to obtain an estimate of the size of the fig) based on the formula (ovoid surface in mm^2^ = [(Diam1/2)*(Diam2/2)*π]. This surface calculation, an approximate of the size of figs, is called "size" hereafter. After opening each fig, we first confirmed whether the fig was pollinated or not by observing the presence of darkened floret styles within the figs. We then recorded the presence, identity, and number of pollinating wasps.

### Statistical analysis

#### Morphospecies coexistence

Firstly, we assessed whether the two morphospecies compete for access to the figs of a tree. Interspecific competition for available, receptive figs will translate into the presence of one species reducing the probability that the other species will visit the fig. We tested this probability using contingency 2x2 tests.

Because individual fig receptivity can last several days but will stop a few hours after sufficient visitation [24], the distribution of foundresses across figs should reflect this process as visited figs rapidly become non-attractive, even closing their ostiole. If choosing to enter a receptive fig is random, and figs lose attractivity with increased visitation, then the distribution of both morphospecies in the crops of *F*. *septica* should be close to a zero-truncated Poisson distribution, with most figs being visited by a low number of foundresses and a decrease in the number of figs visited by increasing numbers of foundresses. We calculated the value λ (the mean number of events within a given interval of time or space, with events being the number of figs containing one to *n* foundresses) for each crop and fitted each observed sample distribution to a theoretical Poisson distribution.

Several predictions can be made related to the variation in fig availability and asynchronous fruiting phenology of *Ficus septica*. First, if the density of surrounding *F*. *septica* trees is sufficient, enough receptive figs should be available for colonisation, and a random distribution similar to a Poisson distribution should be observed. Second, in the case of low availability of receptive figs (due to low tree density in the surroundings and unfavourable climatic conditions) or close vicinity of a receptive fig for emerging pollinators, clumping of foundresses should be observed, and the observed distribution should better match a negative binomial distribution [29]. Finally, in the case where pollinators can recognise the presence of a foundress in a fig and avoid it, a deficit in the number of figs containing two or more foundresses should be observed. Therefore, the observed distribution should also be statistically different from a Poisson distribution.

To better understand how each morphospecies chooses to enter a fig or not, we counted the number of figs containing *x* foundresses, with *x* varying from one up to the greatest number of foundresses observed. Zero-truncated distributions were chosen despite the possibility of having zeros as only one of the morphospecies seems to re-emerge. We then obtained distributions for each sampling period for the following four categories: black morphospecies alone, orange morphospecies alone, black morphospecies with (in the presence of) orange morphospecies, orange morphospecies with black morphospecies. For each sampling period, we calculated the value of λ that maximises the likelihood of the observed data and fitted each distribution against a Poisson theoretical distribution (negative binomial distributions were not used here due to too small samples). Finally, to assess the possible influence of one morphospecies on the other, we also compared the distributions of cumulative (over the entire survey) number of figs containing one-to-*n* foundresses for each morphospecies alone and in the presence of the other morphospecies using Mann-Whitney tests.

#### Fig size

We also investigated whether the size of the recently pollinated figs was constant between male and female trees, across and within individual trees, and between dates of observation for the same tree. Due to our small sample size and potentially non-normal distribution of our data, this preliminary investigation was conducted through non-parametric tests (Mann-Whitney, Kruskal-Wallis, and Friedman tests).

#### Decision trees

Finally, we developed machine learning classifiers to assess the relative abundance of the two morphospecies of pollinating wasps. The decision trees were built in R v3.6.3 [36] using the RPART [37] and RATTLE [38] packages. Two types of decision trees were developed: regression trees for the continuous variables (e.g., the number of foundresses found in figs) and classification trees for the categorical variables (e.g., presence/absence of the two morphospecies).

Decision tree models are based on splitting our target data, the number of wasps per fig, according to the following explanatory variables: tree sex, tree individual, date of observation, season, number of foundresses belonging to the other morphospecies, and fig size. Decision trees are created by processing data from a root node to the leaves. Data is then repeatedly split into branches according to the predictors’ importance and purity level (how homogeneous the subsets of data after the split are). Using reduction of variance (F-test) with P < 0.05 as the split criterion [39], the variables and nodes that best divide our target variables into sub-datasets were identified and selected. To avoid overfitting, we further trained and selected our decision tree models by generating 500 decision trees and ranking them through the random forest method [40]. Random forests generate a high number of decision trees (here: 500 trees) to assess the importance of each variable through the total decrease in node impurities using the Gini Index averaged on all trees. In other words, the impurity is calculated according to how homogeneous the splits are. We used the RANDOMFOREST package [41] to obtain robust estimates of the importance of the variables. Default parameters were used in both decision trees and Random Forest analyses.

## Results

Out of the 673 figs collected, 78.8% (530 figs) were pollinated (presence of darkened floret style), and 21.2% (143 figs) were not. Of the 530 pollinated figs (407 male and 123 female figs), 90% (467 figs) contained at least one foundress, while 12% (63 figs) had none.

### Re-emergence

Observations in the field suggest that the absence of foundresses in these figs with darkened floret styles may be due to re-emergence, or secondary dispersion, events. For example, one orange morphospecies foundress was observed wingless and walking outside a recently pollinated fig being sampled in October 2019. Another foundress of the same morphospecies was observed exiting a pollinated fig in March 2020 (Fig 2). Finally, out of the 63 pollinated figs without foundress, 14 (22.2%) were collected on female trees and 49 on male trees. The absence of significant difference across sex in the number of pollinated figs containing at least one foundress (male = 358 and female = 109) and the pollinated figs without a foundress (male = 49 and female = 14) indicate that this secondary dispersion behaviour is similar in both male and female figs (Pearson Chi-square = 0.39, *p* = 0.84).

### Morphospecies coexistence and distribution

In this study, 38.1% (n = 178) of the figs contained both species of pollinators, 47.5% (n = 222) had only the orange morphospecies, 14.3% (n = 67) had only the black morphospecies, and 21.25% (n = 143) were not pollinated. There was a significant difference in the distribution of the two morphospecies (χ^2^ = 9.09, d.f. = 1, p = 0.003). The result was also significant if adding the 63 pollinated figs without foundresses to the figs containing only the orange morphospecies (only the orange foundresses re-emerge) (χ2 = 27.92, d.f. = 1, p < 0.00001). Comparing expected and observed accounts, there were too few figs with a single species and too many figs with both morphospecies and figs with nothing. Rather than suggesting that the two species seek each other out, this result indicates that fluctuations in the availability of receptive figs in the local environment force pollinator aggregation when the resource is limited and sparsification when the receptive figs abound.

**Fig 3:**
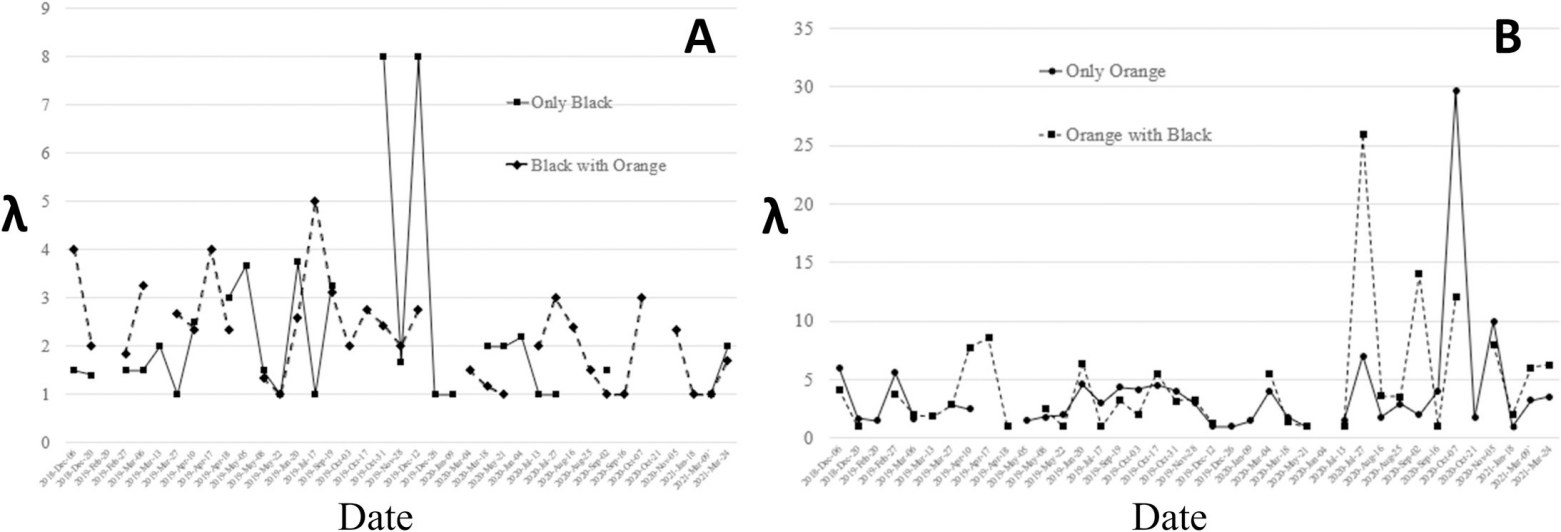
Lambda parameter during the survey for the black (A) and orange (B) morphospecies.

We identified and counted 592 individuals of the black morphospecies and 1828 individuals of the orange morphospecies. The average number of foundresses in pollinated figs containing at least one pollinating female wasp was 5.18 ± 6.49 (Table 1). The most common number of foundresses was one (24.8% of pollinated figs) or two (19.5% of pollinated figs). The number of figs containing foundresses decreased with a higher number of foundresses (S2 Fig). While we observed a significant number of figs containing ten or more wasps (n = 68, S2 Fig), the distribution patterns from our survey samples suggest that wasps cannot determine the number of foundresses before entering a fig. The average value for crops of the black wasps was λ = 2.0 (Fig 3A), while the average value for crops of the orange morphospecies was λ = 4.5 (Fig 3B). These values were the same when considering the morphospecies alone or in the presence of the other morphospecies. However, the orange morphospecies has a re-emergence rate of around 12%, so the λ value is likely slightly underestimated due to undercounting. None of the per-census distributions for the four categories (black morphospecies alone, orange morphospecies alone, black morphospecies with orange morphospecies, orange morphospecies with black morphospecies) deviated from a Poisson distribution indicating randomness in the choice of entering figs. Additionally, there was no significant difference in the distribution patterns of one morphospecies alone or in the presence of the other morphospecies in the survey-cumulated distribution [sum of all per-census distribution] (Mann-Whitney tests: black morphospecies, p = 0.400; orange morphospecies, p = 0.529).

**Table 1:** Distribution of orange and black morphospecies distribution in male and female pollinated figs.

	Figs with both morphospecies			Figs with one morphospecies	
	All	Orange	Black	Orange	Black
**All figs (N = 477)**	5.18 ± 6.49	4.57 ± 6.44	2.42 ± 2.11	4.18 ± 6.41	2.30 ± 2. 28
	1–60	1–60	1–15	1–60	1–14
**Male figs (N = 358)**	5.42 ± 7.05	4.81 ± 6.97	2.31 ± 2.53	4.48 ± 6.97	1.85 ± 1.63
	1–60	1–60	1–15	1–60	1–11
**Female figs (N = 109)**	4.40 ± 4.04	3.66 ± 3.60	2.69 ± 2.26	2.88 ± 2.64	2.96 ± 2.91
	1–19	1–17	1–14	1–10	1–14

The values are displayed as average ± standard deviation and range

However, the cumulated distribution of the black morphospecies was significantly different from that of the orange morphospecies, whether alone (Mann-Whitney test, z = 2.811, p = 0.005) or in the presence of orange individuals (Mann-Whitney test, z = 2.773, p = 0.006). This difference is likely due to the lower density of black wasps in our study site.

### Fig size

The size of the 143 unpollinated figs (mean ± standard deviation: 186.09mm^2^ ± 40.59) was significantly smaller than the size of the pollinated figs (207.33mm^2^ ± 39.48; independent sample t-test, t_671_ = -5.674, p < 0.001). These 143 unpollinated figs were not used in further analyses as no wasp entered them.

The pollinated figs without foundress (220.51mm^2^ ± 33.51) were significantly larger than those containing at least one foundress (205.55mm^2^ ± 39.92; independent sample t-test, t_528_ = -2.842, p = 0.005). It is likely that these figs without foundress were collected at the end of their receptivity phase or had already passed it, which could explain their larger size. These figs were also not used in further analyses. Among the 467 pollinated figs containing at least one pollinating wasp (358 male and 109 female figs), fig size was not significantly different between trees of different sexes (Mann-Whitney test, mean ranks: males = 230.85 and females = 244.34, U = 20,638, *p* = 0.36 two-tailed). After selecting across the whole survey, the census samplings with the highest number of figs for the same tree, we removed all other measures taken on the same trees to remove any time effect. We then compared the size of the figs between individual trees. We found that the size of the pollinated figs significantly varied across individual trees (Kruskal-Wallis test, KW = 71.77, p<0.001).

We found slight variation in fig size for the same tree over time. Three of the 15 tested trees that were censused between two and fifteen times each showed significant differences in fig size over time. In the first tree, one series of 10 values was significantly smaller out of five censuses (Tree ID007—Friedman’s two-way analysis of variance, p = 0.011). The other two trees with significant differences had only two censuses each: Tree ID25—Wilcoxon signed-rank test: p = 0.007; Tree ID199—Wilcoxon signed-rank test: p = 0.012). All other trees with equal or higher numbers of censuses showed no significant difference over time. In total, only three censuses out of the 15 trees and 63 censuses showed significant variation. This suggests that *F*. *septica* trees produce similarly sized figs throughout the year.

### Regression trees

**Fig 4:**
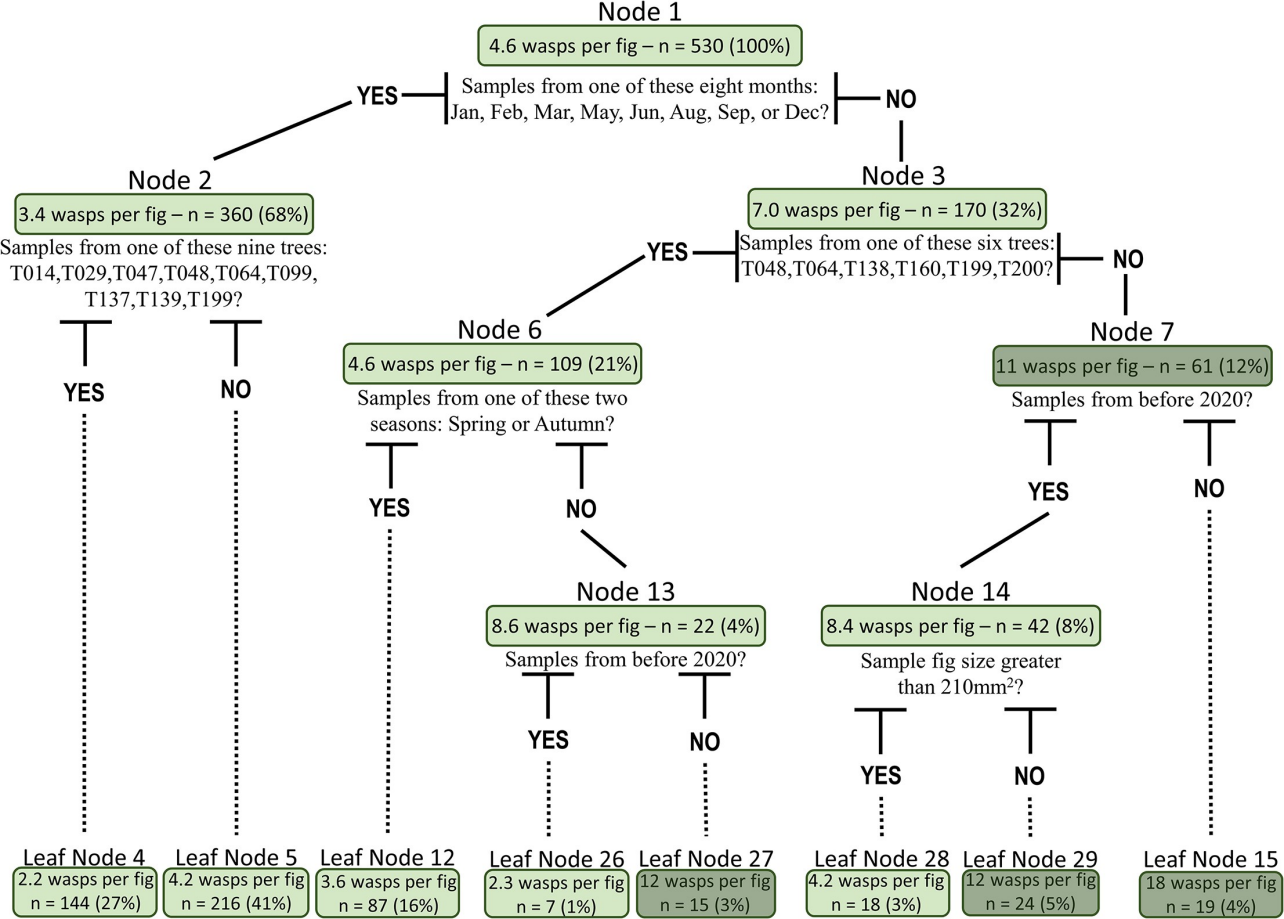
Regression tree for the average number of wasps per fig. At each node, the tree divides the samples based on the question indicated below the node. The node information consists of five components: Node number, average number of wasps at this node, number of samples (in this figure, number of figs), percentage of the samples from the total number of samples at this node, and the question that divides the samples into two groups. Nodes coloured in dark green are for nodes where the average number of wasps is greater than ten.

**Fig 5:**
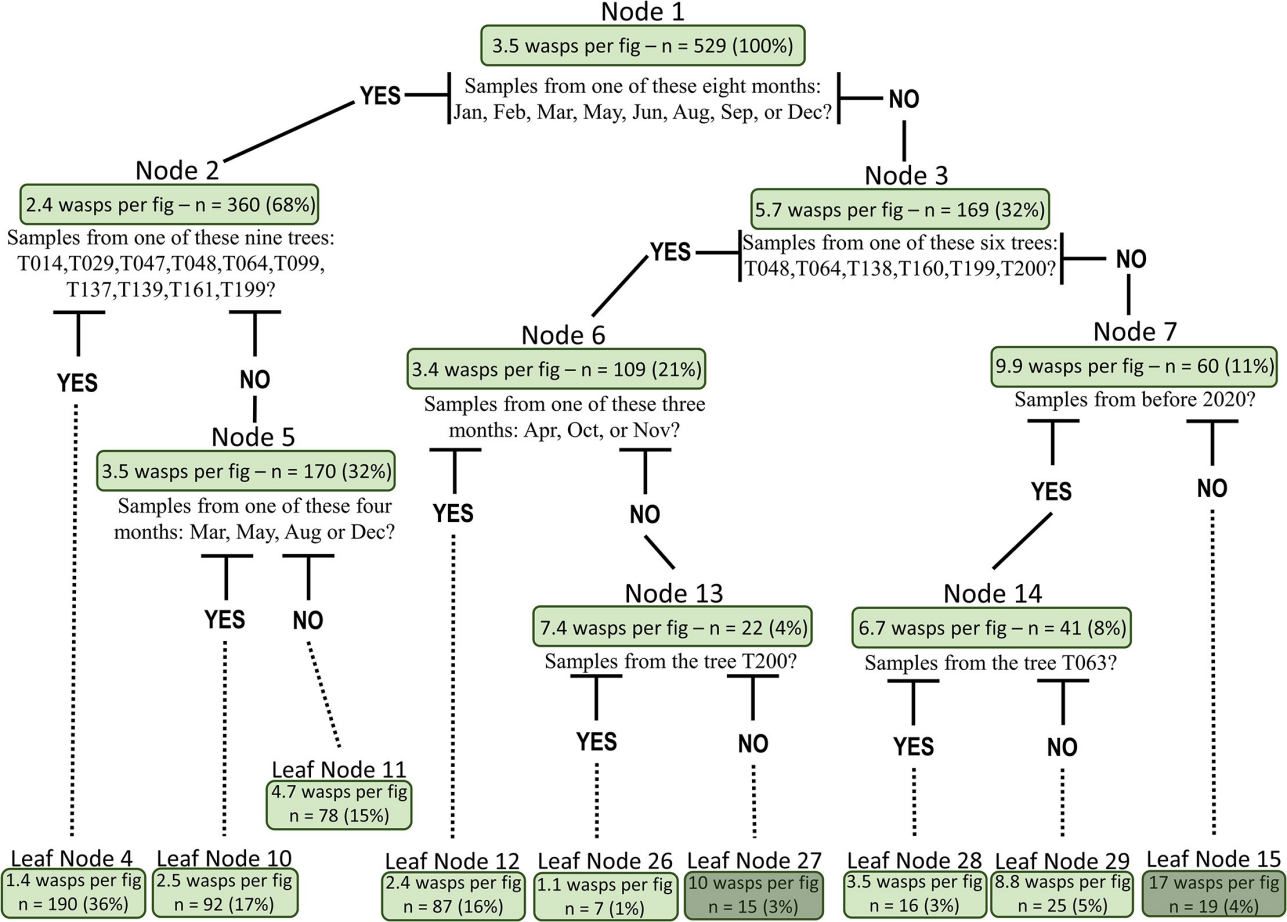
Random forest plot of variable importance for all the pollinators pooled together. These plots rank the effect of different variables; the higher the number, the more important the variable. The values of IncNodePurity on the X-axis are averaged from the total decrease in node purity. The node purity is calculated from the variable values at a split using the Gini Index [42]. The Y-axis labels: SIZE is for the size of the fig, MONTH is for the month when the fig was collected, ID is for the tree that the sampled fig came from, YEAR or SEASON is from when the fig was sampled, and SEX is for the sex of the tree of the collected fig.

Regression trees (decision trees) were used to classify the factors that affect the total number of pollinators within a fig. The trees showed seven significant splits and emphasised the importance of the sampling month and tree identity (Fig 4). However, when using the same dataset, a random forest analysis found that fig size was the most important variable, followed by the sampling month and tree identity (Fig 5). The regression tree for the total number of orange morphospecies followed a similar pattern to the overall number of wasps (S3 Fig), but the total number of black wasps was more associated with the identity of the tree (S4 Fig). In contrast to the regression trees in S3 and S4 Figs, the random forest analysis identified fig size as the most important variable for both morphospecies (S5 and S6 Figs). The same set of explanatory variables influenced the abundance of the combined morphospecies and the abundance of orange wasps. In contrast, the abundance of black wasps was mainly affected by the identity of the considered tree. The abundance of the other morphospecies came fourth in both random forest variable importance plots (S5 and S6 Figs). Tree sex was consistently classified as least important in these analyses.

**Fig 6:**
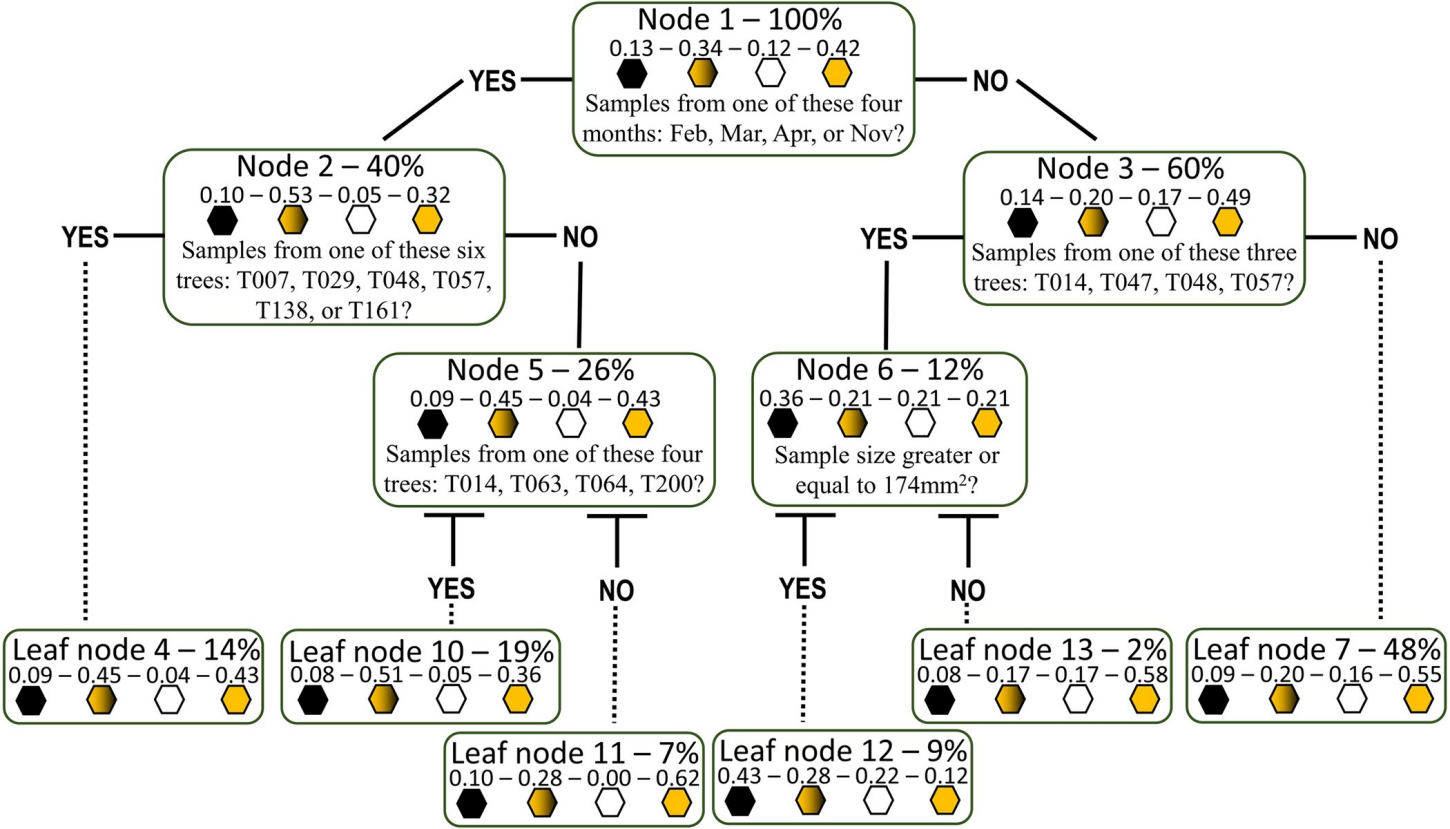
Classification tree for the presence of the two morphospecies (for the plots showing the mean decrease in Gini of the presence of the two morphospecies, see S7 Fig). The proportions of the categories are shown on the top of the coloured hexagons: Black for the figs that contain only black wasps, black and orange for the figs containing both morphospecies, white for figs containing no wasp, and orange for figs that contain only orange wasps.

Classification trees were built using four categories: both morphospecies simultaneously present, only orange wasps, only black wasps, and none (empty pollinated figs) (Fig 6). The first node greatly divided the population using a question about the sample month: On one side, 53% of the figs had both species of wasps, and on the other side, 20% of the figs had orange and black wasps (Fig 6). For the categorical random forest, fig size was more important than any other variables (S7 Fig). A final classification tree was built using four other categories according to the most abundant wasp morphospecies in each fig: orange wasps, black wasps, none, and the same number of wasps (S8 Fig). The random forest importance plot was similar to others (S9 Fig). Still, the classification tree displayed fig size and tree identity as its two first splits.

## Discussion

In this study, we used a longitudinal survey to assess pollination mechanisms in *Ficus septica* figs over two years. We tested four assumptions: the one-to-one rule, no re-emergence, low foundress number, and random settlement. Our results showed that only the assumption of random settlement was valid. We found that (i) two species of pollinating wasps cohabit in the figs of *Ficus septica*, (ii) the orange morphospecies re-emerges after ovipositing, (iii) receptive figs contain an average of five foundresses, and (iv) female pollinators randomly enter receptive figs. Over 75% of the figs had several foundresses, and more than a third held both morphospecies simultaneously. We found no evidence of aggressive interference competition (attacks or bites) between the two morphospecies sharing the same receptive figs. Instead, our data suggest that ecological factors drive the local abundance of foundresses. Indeed, the sampling period, fig size, and tree identity were the best predictors of the presence and higher number of foundresses.

### Foundress re-emergence

The assumption that foundresses perish in the fig they have oviposited was true only for the black morphospecies. Indeed, we observed that 10% of pollinated figs contain no foundress and several wingless orange foundresses crawling out and walking on the figs of *F*. *septica*. While some females may exit and die on the surface of the figs they have pollinated, others sometimes enter and oviposit in another fig. While our data confirm re-emergence, whether secondary oviposition follows remains unknown. Our finding is consistent with observations in other *Ficus*-pollinator systems where foundresses pollinate and oviposit in several figs [13]. Re-emerging foundresses, which can pollinate and oviposit in multiple figs, may have a selective advantage over foundresses that die in the first fig they enter because secondary oviposition can increase pollination [13, 15, 16], minimise the risk of losing all their offspring in one fig by spreading out their eggs [16], reduce competition for oviposition sites [43], and decrease the probability of inbreeding among siblings (e.g., in *F*. *montana*, [16]; in *F*. *deltoidei* [44 but see 45]). On *F*. *septica*, the re-emergence behaviour may have been favoured by the peculiar phenology of this fig species, which often has another receptive fig available at the same node or a few distance nodes on the same branch (S1 Fig). It is puzzling that no re-emergence was observed for the black morphospecies. A slightly bigger size of the black morphospecies, which increases the required efforts and damages sustained while entering the ostiole, might explain this discrepancy. Further research on the frequency of re-emergence and possible mechanisms of secondary pollination is needed, as re-emergence and secondary pollination may be more common than previously assumed.

### Foundress cohabitation with random pollination

Because the number of foundresses limits the windows of receptivity of the figs [24] and wasp oviposition [23, 27–32], we expect foundresses to exhibit behaviours allowing them to optimise their reproduction and minimise that of other foundresses. However, we did not find evidence of interference competition between the two morphospecies or between individuals of the same morphospecies within figs of *F*. *septica*. This contrast with other studies that have reported competitive avoidance [9, 11, 22], aggressive interspecific [27, 28] or intraspecific [31] competition. While our data do not suggest any aggressive behaviour, other studies [27, 28, 46] have observed interferences during oviposition. Because our study primarily uses foundress distributions, we cannot rule out the possibility that interference affects the oviposition rate. To confirm that *Ceratosolen* wasps of *F*. *septica* are not involved in any contests or fights, further observations of the behaviour of living foundresses over longer periods are needed. Our study found that, on average, each fig of *Ficus septica* contained five foundresses, and 38% of the figs had both morphospecies. This is the highest level of species cohabitation reported in the literature to date. Other studies [7, 8, 47, 48] have also shown that *Ficus* figs can be pollinated by multiple wasp species, contradicting the previous assumption of a single pollinating species. The high number of foundresses in our study, coupled with the lack of evidence for interference competition, suggests that the large number of ovules in *Ficus septica* figs may not limit pollinator ability to oviposit. This explanation is consistent with Dunn et al. [28, 43] findings on *Ficus citrifolia* in Panama and Brazil. However, further research is needed to confirm this hypothesis, including a thorough examination of the number of wasp offspring for different foundress numbers, as well as the number of eggs per foundress in relation to the number of flowers and the mean foundress.

As expected, the frequency of figs containing one or two foundresses was high and decreased with a higher number of foundresses (Fig 3). These per-census distributions of figs containing an increasing number of foundresses did not deviate from a Poisson distribution, a result similar to those observed in the crops of *F*. *salicifolia* [29]. These Poisson-like distributions support the idea that entering a fig is a random choice for female pollinators, who enter the first available fig they find [33]. Additionally, the distribution patterns from our survey samples indicate that wasps do not avoid figs that have already been visited. Interestingly, we observed many figs containing ten or more wasps (n = 68) and up to 60 foundresses within a single fig, a remarkable number considering the size of the fig itself. These numbers are likely underestimated as orange individuals that re-emerge may have left the fig before our census. Nevertheless, this is far above the number of foundresses (less than ten) reported in the smaller figs of *F*. *salicifolia* [27] and *F*. *rubiginosa* [20], as well as in the similar-sized figs of *Ficus hirta* [49], and in the bigger figs of Brazilian *F*. *citrifolia* [50] and South African *F*. *sansibarica* [51]. Still, Greeff et al. [22] reported up to 23 foundresses in the similar-sized figs of *F*. *sycomorus*, and Wang et al. [21] found more than 80 foundresses in the larger figs of *F*. *racemosa*.

The high numbers of foundresses observed in our study are surprising, as there is a cost associated with sharing the same receptive fig. Greeff & Newman [29] suggested that ecological factors such as the close proximity between emerging and receptive figs and prevailing winds in the area could be responsible for clumped distributions. In the crops of *F*. *septica*, the five most populated figs were found in two crops: One in October and one in July, which are low-production months for the *F*. *septica* male trees (i.e., after the spring crop peak and after the end of summer peak respectively [52, 53]). This suggests that the number of wasps per fig may be influenced by the total number of receptive figs available in the environment and thus, the phenological cycle of the fig tree.

### Environmental factors and phenology mediating pollinator-fig interactions

A critical factor explaining the abundance of foundresses is the tree identity. Regardless of the sampling season or period, certain trees harboured a much greater number of foundresses than others. Factors intrinsic to the tree or the fig may lead to these differences.

First, the fig size (Fig 5, S5 and S6 Figs) is the most important variable according to all the Random Forest analyses. Larger figs contain more ovules available for oviposition [54], but they also have larger ostioles (the opening at the base of the fig) and lumens (the empty space at the centre of the fig), which makes it easier for pollinators to enter and aggregate in these figs. The larger ostioles and lumens allow for greater foundress clumping within a fig, but they also result in larger galls that provide better mating opportunities [55], better survival [56], and a lower predation rate [57]. While our study found that foundresses do not choose their figs, it is possible that larger figs size are more attractive to pollinators due to the production of a larger amount of volatile organic compounds (VOCs). Indeed, the amount of emitted VOCs is correlated with the size of the fig [58]. Another important characteristic of the receptive fig is its gloss, which reflects light. To our knowledge, this gloss has not been previously studied, as even taxonomic studies do not mention it [34]. Larger figs may reflect more light than smaller figs and therefore be more attractive to searching pollinators. After pollination, figs quickly lose their gloss along with the olfactory cues signalling receptivity [26]. As fig size varies between *F*. *septica* trees but remains somewhat constant over the year, it is likely that individual trees producing the largest, and therefore most attractive, figs will also attract a greater number of pollinators.

Second, the local environment may significantly affect the number of foundresses. As the foundresses do not choose figs, the distance between trees (tree density) and the “visibility” of a tree in the environment may be critical. For example, the five most populated figs in this study came from two trees separated by an open area, including a volleyball field and a small stream, making them highly visible to pollinators. Additionally, this “visibility” might also be related to the unique blend of olfactory cues emitted by a receptive fig [2], as the signal may travel easily in such an open environment. Another variation in the local environment is the window of receptivity. Older receptive figs have been associated with a greater reluctance for pollinators to enter the fig ostiole [59]. Local variations in the availability, age, and level of attraction of receptive figs may, in turn, create variability in foundress numbers.

Another critical factor affecting the abundance of the foundresses identified by machine learning was the time (year, season, and months). The time of the year is susceptible to climatic conditions, which can affect pollinators. Low humidity levels [33] and high temperatures [60, 61] can directly stress pollinating wasps and shorten their lifespan. Interestingly, Wang et al. [46] showed that in *Ficus racemosa*, environmental conditions lead to fig under-exploitation in the summer (few foundresses, few seeds, few galls, and many empty ovules) and fig over-exploitation in the winter (many foundresses, many seeds, many galls, and few empty ovules). These direct effects through differences in the temperature tolerance of fig wasps help maintain the pollinating wasps appropriate symbiotic behaviour and stabilise the fig-fig wasp mutualism [46] and are a critical factor in maintaining interspecific interactions and coexistence [62]. In a study about the daily rhythm of fig pollinators, Conchou et al. [63] observed that the black and orange morphospecies that pollinate *F*. *septica* in the Philippines were active at the same time. However, the black wasps could be active for a few more hours later in the day. Such differences in temperature tolerance may locally provide some selective advantages to certain species over others. Because in our study area, both morphospecies are at the limit of their geographical distribution (northern limit for the orange wasps and southern limit for the black wasps), variations in the *Ficus*-fig wasp interaction are expected [46]. Therefore, further studies should focus on comparing foundress distributions found in more standard ecological niches of these two morphospecies. This will allow us to assess the pollination mechanisms without other pollinating species.

The time of year also indirectly affects pollinators, as meteorological factors (temperature, rainfall, and sunshine hours) influence fig production and maturation [53, 64], and thus receptive figs availability. In *F*. *septica*, fig production strongly fluctuates over time and across regions in Taiwan [52, 53]. For instance, in northern Taiwan, female trees show a homogenous low production throughout the year, while male trees show two production peaks in April and September. In our regression trees (Fig 4, node 1, for example), the winter months (December to March) and a few other months were associated with a greater number of foundresses. These months are usually associated with low fig production in the phenology of *F*. *septica* [52, 53], possibly forcing female pollinators to cluster within the few receptive figs that are available. Other studies have identified similar peaks in foundress abundance (e.g., in December, March and April in *F*. *salicifolia* [29] and pollinator abundance (e.g., in summer in *Ficus microcarpa* [65]). Changes in pollinator survival have also been linked to seasonal environmental conditions [62]. However, such peaks in abundance and survival variation were not associated with receptive fig availability. We propose that in *F*. *septica*, frequency-dependent interactions between fig production and maturation and pollinator tolerance to local environmental conditions could mediate the abundance of foundresses in the figs. We hope that future studies will untangle the respective roles of such extrinsic and intrinsic factors on the number of foundresses, seeds, and pollinators produced in the figs of *F*. *septica*.

### Studies limitation

Approximately one-fifth of the collected figs were not pollinated, demonstrating the difficulty of distinguishing between pollinated and unpollinated figs based on their external appearance. It was also challenging to consistently visit the study field during the Covid-19 pandemic, which may have resulted in overlooking some local factors affecting foundress distribution. Additionally, when the receptivity phase ends, the fig swells, which can cause the destruction of the remains of the foundresses and result in an inaccurate foundress count [43]. Further research comparing fig wasps and tree environmental diversity at a finer geographical and temporal scale, as well as the role of local physical factors affecting them, is needed. As tree density may also play a role in concentrating or diluting the foundress number within a crop, we encourage further research using study fields with different densities of *F*. *septica* trees to clarify this relationship.

## Conclusion

Our study provides insights into a fundamental aspect of pollination biology: flower choice. We found that there is no choice, and female pollinators seem to enter the first receptive fig they encounter, indicating that fig wasps may be guided by simple stimuli. As a result, three out of the four assumptions mentioned in the introduction of this study (one-to-one rule, no re-emergence, low foundress number) have been rejected. Only the assumption of random settlement has been confirmed. Furthermore, our findings indicate that the sampling period and the identity of the tree were the prime predictors of the presence and number of foundresses within the figs, emphasizing the significance of the tree local environment. At last, the occurrence of two pollinating species within such a restricted part of the *F*. *septica* distribution in Southern Taiwan warrants exciting future studies on the mechanisms of pollinator coexistence and their distinct impacts on their mutualistic host.

## Supporting information

S1 Fig Branch of *Ficus septica* with figs at different developmental phases. (TIF)

S2 Fig Frequency histogram showing the number of figs for ranked numbers of foundresses found inside the figs. The white bars represent the values for both species taken together, while the orange and black bars represent the values for the sole orange morphospecies and the sole black morphospecies. The enlarged graph shows frequencies for pollinator numbers ranging between 33 to 60 foundresses (no foundress value between 28 and 32).
(TIF)

S3 Fig Regression tree for the average number of orange wasps per fig. For a complete explanation of the tree, please refer to Fig 4. Nodes coloured in dark green are for nodes where the average number of wasps is greater than ten wasps. The plot of variable importance is on S5 Fig.
(TIF)

S4 Fig Regression tree for the average number of black wasps per fig. For a complete explanation of the trees, please refer to Fig 3. Nodes coloured in yellow are for nodes where the average number of wasps is lower than one wasp, in orange for nodes lower than 0.1 wasps. The plot of variable importance is on S6 Fig.
(TIF)

S5 Fig Random forest plot of variable importance for the orange wasps. See Fig 5 for the X-axis (IncNodePurity) values calculation. SURF stands for the size of the figs and BLA for the black morphospecies.
(TIF)

S6 Fig Random forest plot of variable importance for the black wasps. See Fig 5 for the X-axis (IncNodePurity) values calculation. SURF stands for the size of the figs and ORA for the orange morphospecies.
(TIF)

S7 Fig Plots show the mean decrease in Gini of the presence of the two morphospecies related to Fig 6. Parameters are sorted according to their importance.
(TIF)

S8 Fig Classification tree for the most abundant morphospecies (for the plots showing the mean decrease in Gini of the most abundant morphospecies, see S9 Fig). The proportions of the categories are shown on the top of the coloured hexagons: Black for the figs that contain only black wasps, black and orange for the figs containing both morphospecies, white for figs containing no wasp, and orange for the figs that contain only orange wasps.
(TIF)

S9 Fig Plots show the mean decrease in Gini of the most abundant morphospecies related to S8 Fig. Parameters are sorted according to their importance.
(TIF)

S10 Fig Frequency histogram showing the number of figs for ranked numbers of foundresses. Only figs containing a single morphospecies are considered. The orange and black bars represent the number of figs for the orange and black morphospecies. The enlarged graph shows frequencies for pollinator numbers ranging between 39 to 60 foundresses (no foundress value between 25 and 38).
(TIF)
