# Supplementary figures and images for: Local ecological factors, not interference competition, drive the foundress number of two species of fig wasp sharing Ficus septica figs
Source: PLoS One. 2024 Jan 2;19(1):e0290439. doi: 10.1371/journal.pone.0290439 (PMC10760673; doi:10.1371/journal.pone.0290439)

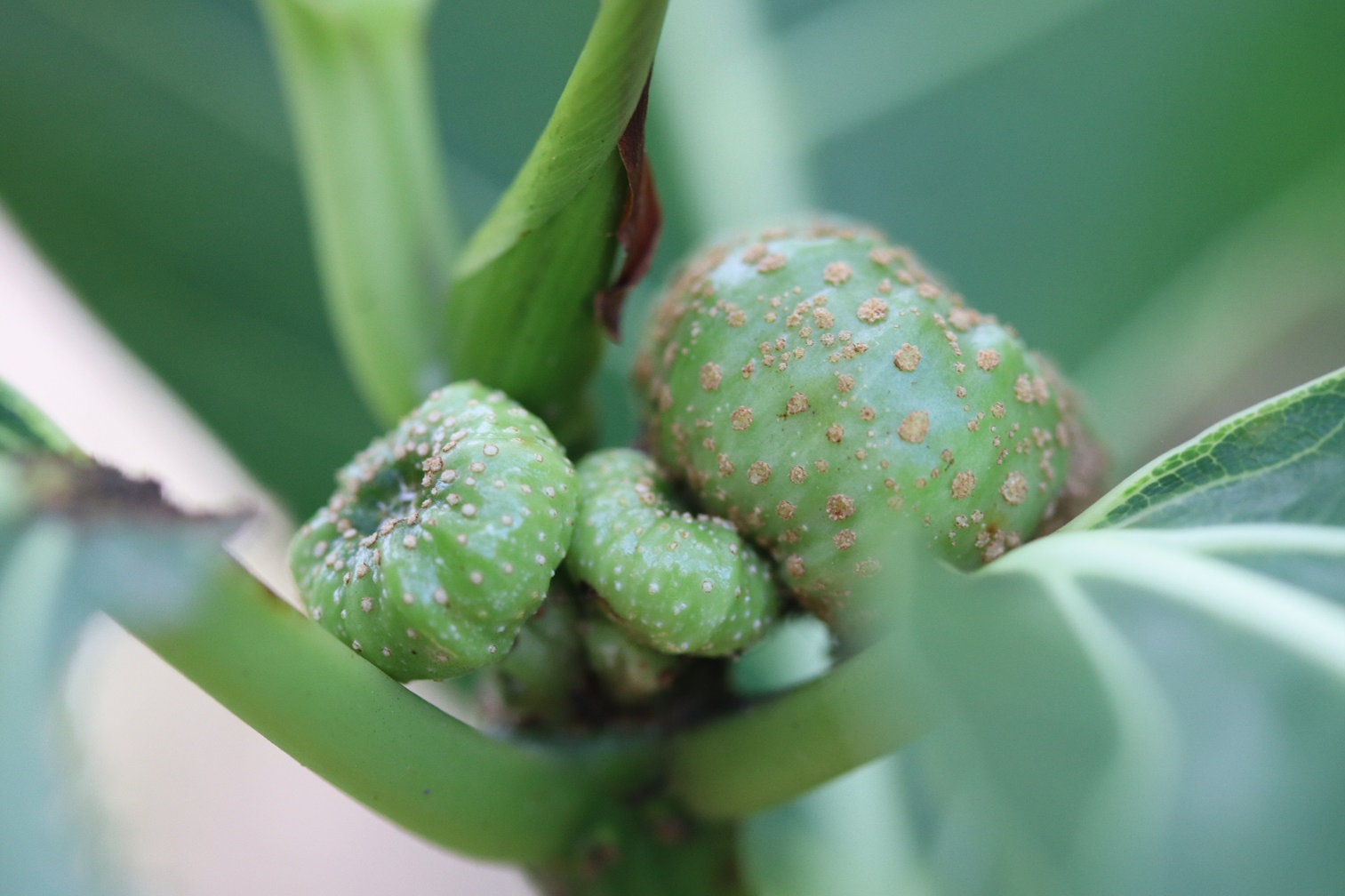

Supplement: S1 Fig — (TIF) [file pone.0290439.s001.tif]

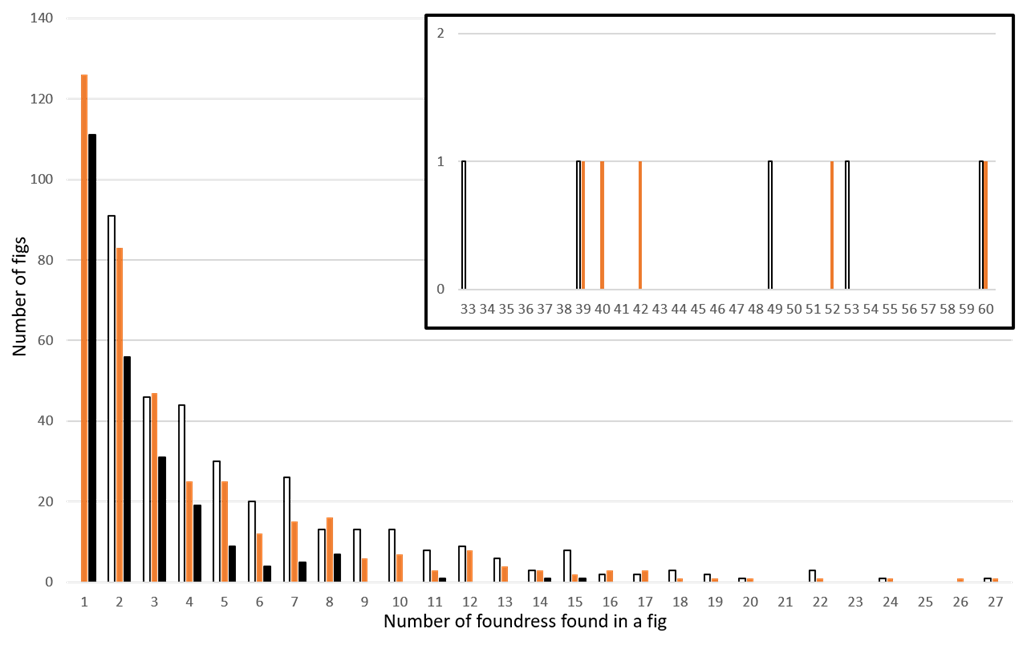

Supplement: S2 Fig — The white bars represent the values for both species taken together, while the orange and black bars represent the values for the sole orange morphospecies and the sole black morphospecies. The enlarged graph shows frequencies for pollinator numbers ranging between 33 to 60 foundresses (no foundress value between 28 and 32). (TIF) [file pone.0290439.s002.tif]

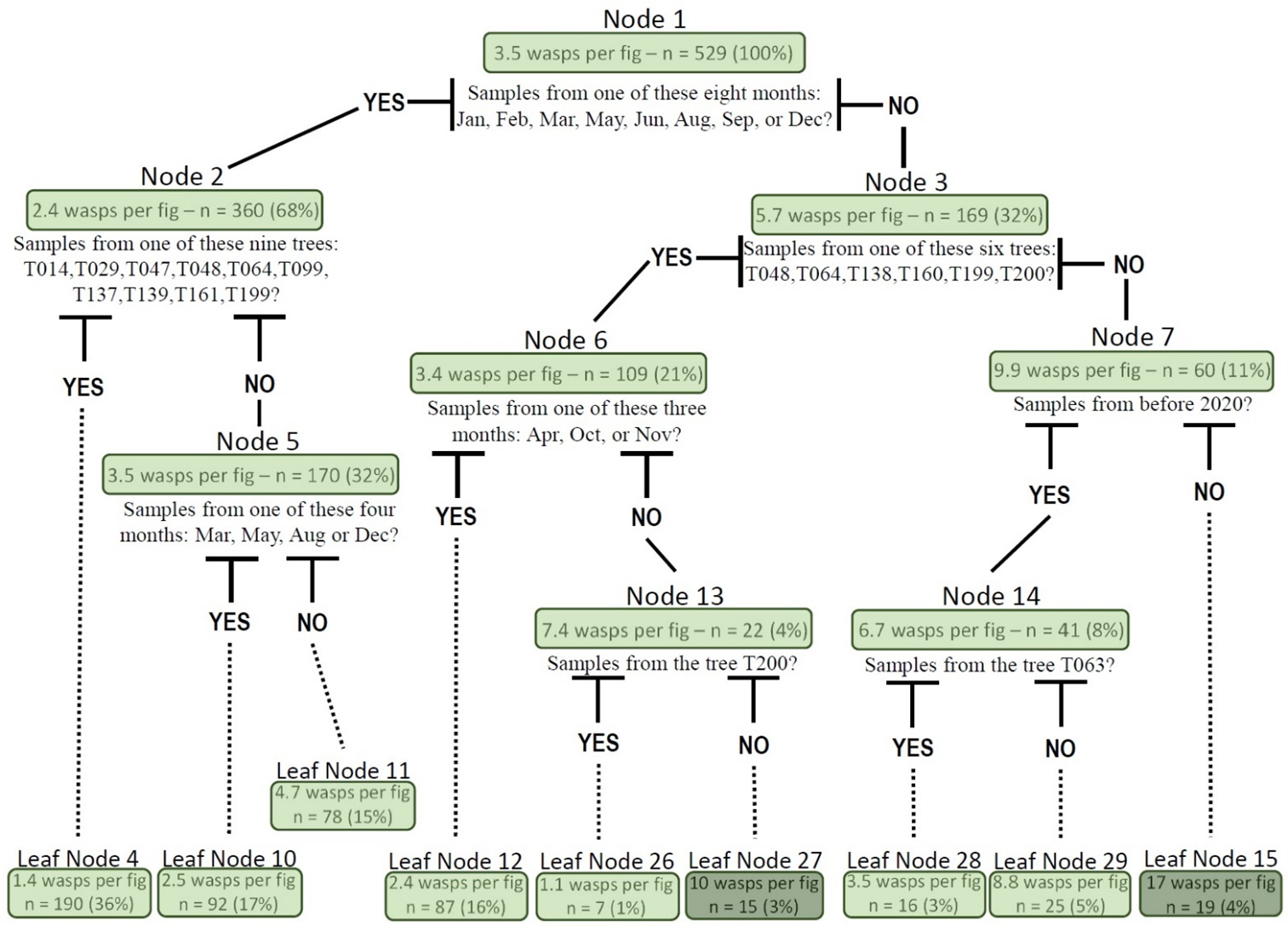

Supplement: S3 Fig — For a complete explanation of the tree, please refer to Fig 4. Nodes coloured in dark green are for nodes where the average number of wasps is greater than ten wasps. The plot of variable importance is on S5 Fig. (TIF) [file pone.0290439.s003.tif]

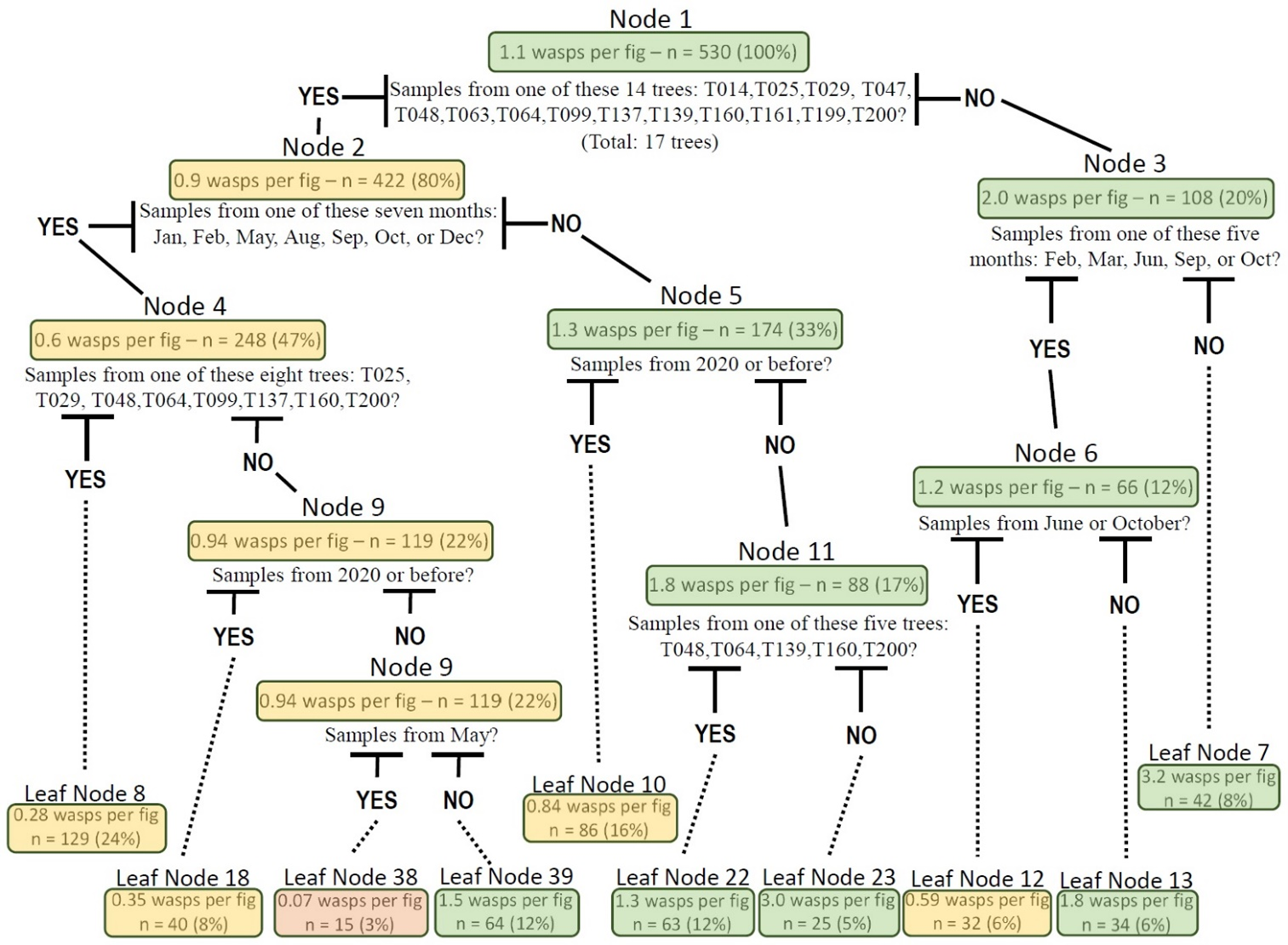

Supplement: S4 Fig — For a complete explanation of the trees, please refer to Fig 3. Nodes coloured in yellow are for nodes where the average number of wasps is lower than one wasp, in orange for nodes lower than 0.1 wasps. The plot of variable importance is on S6 Fig. (TIF) [file pone.0290439.s004.tif]

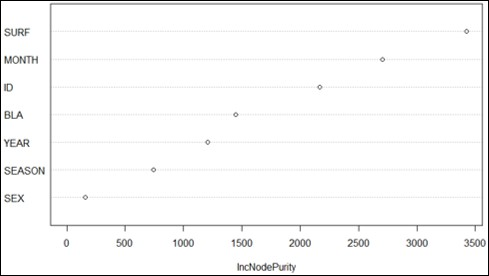

Supplement: S5 Fig — See Fig 5 for the X-axis (IncNodePurity) values calculation. SURF stands for the size of the figs and BLA for the black morphospecies. (TIF) [file pone.0290439.s005.tif]

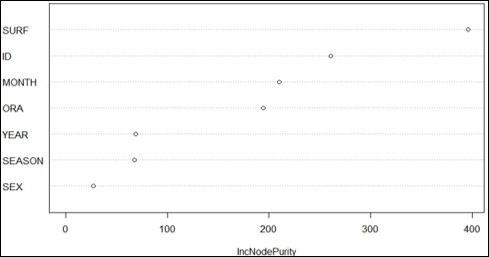

Supplement: S6 Fig — See Fig 5 for the X-axis (IncNodePurity) values calculation. SURF stands for the size of the figs and ORA for the orange morphospecies. (TIF) [file pone.0290439.s006.tif]

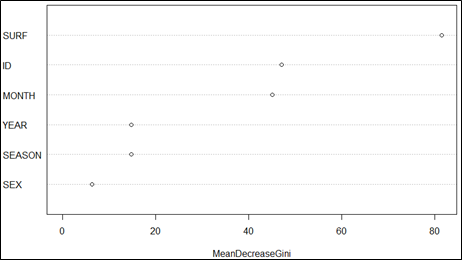

Supplement: S7 Fig — Parameters are sorted according to their importance. (TIF) [file pone.0290439.s007.tif]

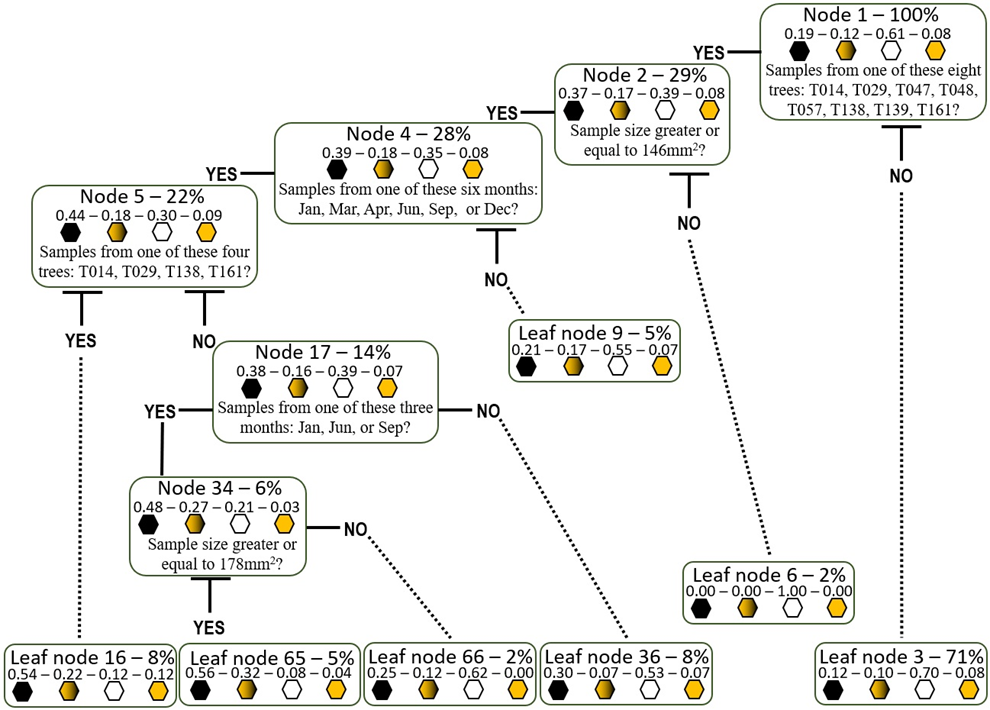

Supplement: S8 Fig — The proportions of the categories are shown on the top of the coloured hexagons: Black for the figs that contain only black wasps, black and orange for the figs containing both morphospecies, white for figs containing no wasp, and orange for the figs that contain only orange wasps. (TIF) [file pone.0290439.s008.tif]

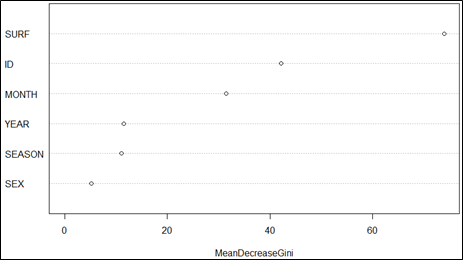

Supplement: S9 Fig — Parameters are sorted according to their importance. (TIF) [file pone.0290439.s009.tif]

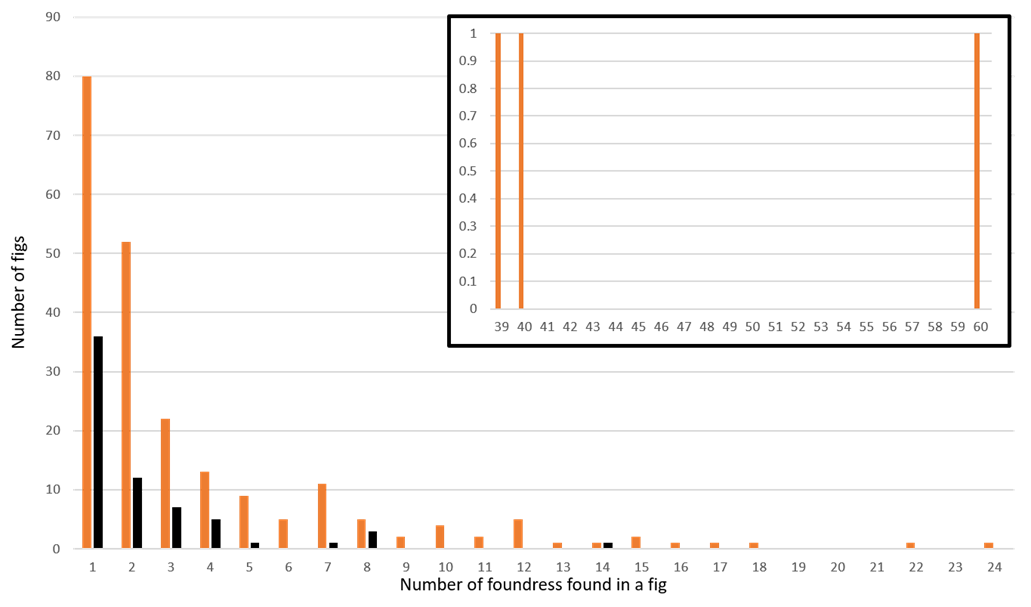

Supplement: S10 Fig — Only figs containing a single morphospecies are considered. The orange and black bars represent the number of figs for the orange and black morphospecies. The enlarged graph shows frequencies for pollinator numbers ranging between 39 to 60 foundresses (no foundress value between 25 and 38). (TIF) [file pone.0290439.s010.tif]
